# Basic Design Parameters Influencing on Axial Stiffness of the Spiral Wound Gasket

**DOI:** 10.3390/ma16186209

**Published:** 2023-09-14

**Authors:** Przemysław Jaszak, Rafał Grzejda, Janusz Kluczyński, Paweł Zmarzły

**Affiliations:** 1 Faculty of Mechanical and Power Engineering, Wroclaw University of Science Technology, Wybrzeze Stanislawa Wyspianskiego St. 27, 50-370 Wroclaw, Poland; 2 Faculty of Mechanical Engineering and Mechatronics, West Pomeranian University of Technology in Szczecin, 19 Piastow Ave., 70-310 Szczecin, Poland; rafal.grzejda@zut.edu.pl; 3 Institute of Robots & Machine Design, Faculty of Mechanical Engineering, Military University of Technology, Gen. S. Kaliskiego St. 2, 00-908 Warsaw, Poland; janusz.kluczynski@wat.edu.pl; 4 Faculty of Mechatronics and Mechanical Engineering, Kielce University of Technology, al. Tysiąclecia Państwa Polskiego 7, 25-314 Kielce, Poland; pzmarzly@tu.kielce.pl

**Keywords:** flange-bolted connection, axial stiffness, spiral wound gasket

## Abstract

The article presents the influence of important design parameters of a spiral gasket on axial stiffness and leakage level. These parameters were the angle of inclination of the central part of the spiral section, the length of the vertical part of the spiral section, and the degree of densification of the material filling the metal coils. The scope of work was divided into two stages. In the first, experimental tests were conducted to determine the stiffness and tightness of a standard spiral gasket at two extreme levels of densification of the filler material, and the elastic–plastic properties of expanded graphite, which is the filler material of the metal spirals, were determined. In the second stage, multivariate numerical calculations were carried out to determine the axial stiffness of the gasket and to evaluate the distribution of contact pressure on the sealing surface. A novel aspect of the work is the proposal of a mathematical model to estimate the averaged value of the modulus of elasticity of the filler material as a function of the degree of densification and the execution of an experimental plan that significantly allowed the adoption of a limited number of analysed model variants used in the numerical calculations.

## 1. Introduction

Spiral gaskets are a type of static seal that is widely used in the flange-bolted connections of medium- and high-pressure industrial pipelines [1,2,3]. Their cylindrical shape and the requirements related to their dimensional and shape accuracy require the development of measurement methods that can be successfully used in industrial conditions [4]. The idea behind the construction of such gaskets is to make a spiral part (which is made of a metal strip) with alternately wound soft filler. The metal strip gives the gasket adequate elasticity and strength, while the filler is responsible for the tightness [5].

Typically, the spiral part is embedded in the inner and outer metal rings [6], the primary function of which is to protect against excessive compression and to prevent the effect of the buckling of the inner winding of the spiral gasket—as presented in [7]. Appropriate embedment of the gasket in the rings and their influence on tightness and stiffness are discussed by Jenco and Hunt [8], as well as by Waterland and Bouzid [9]. The cross-sectional shape of standard design solutions for gaskets of this type involves a serial connection of V-shaped segments. This shape ensures high elastic recovery and relatively high tightness at low contact pressures. The basic geometric relationships describing the dimensions of gaskets used in pressure equipment are defined in ASME B16.20 [10] and EN 1514-2 [11]. These documents describe the method of how they are made. As shown in [12], an alternative to the standard solution may be a gasket with an asymmetric cross-sectional profile, which in turn results in the improvement of essential parameters, e.g., tightness. The type of material that is used to fill the metal windings of a spiral gasket and the density of their winding also have a significant impact on the parameters that affect the quality of such gaskets. This issue was experimentally investigated, analysed, and presented by Veiga et al. [13,14]. Expanded graphite or polytetrafluoroethylene (PTFE) are the most commonly used materials for filling the metal windings of the spiral gaskets [15]. The advantages and disadvantages of their use have been discussed by Hamilton et al. [16]. The use of graphite provides lower contact pressures for the achievement of a higher level of tightness than when using PTFE. However, the tightness of a gasket with PTFE filling can be increased (even by an order of magnitude) by making it with a nonstandard, asymmetric shape of its cross-section, as demonstrated in [13].

It is also worth noting the research on the wear and durability of spiral gaskets. Zhang et al. [17] described the results of frictional wear tests on the filling material, whereas Zhong et al. [6] presented the impact of damage of the retaining rings on the tightness and stiffness of the structure. The results showed that the gasket winding structure affects the variation of the friction coefficient fluctuation.

Many studies of spiral gaskets are devoted to the issues of modelling their operating conditions in flange-bolted connections with the use of the finite element method (FEM) [18,19,20]. In these cases, CAD systems [21,22] are most often used to model the connections. Murali Krishna et al. [23] performed a study on the sealing efficiency of bolted flange connections with spiral wound gaskets using finite element analysis. Jaszak investigated a spiral gasket placed between a pair of hydraulic press plates using a highly compressible elastomeric material model [24] and an elastic–plastic bilinear model [25]. Nelson [26] analysed the stress in spiral wound gasket with graphite filler using a micromechanical approach and axisymmetric finite element analysis. Abid and Hussain [27] and Veiga et al. [28] analysed the effects of using spiral gaskets on a flange bolted connection of a pipeline subjected to multivariate analysis regarding mechanical and thermal loads.

To a more limited extent, methods other than FEM are also used for modelling spiral gaskets. The material behaviour of spiral wound gaskets can be effectively evaluated by remodelling the heterogeneous material through the homogenisation technique [29]. An analytical model of a spiral gasket using the homogenisation method was proposed by Mathan and Siva Prasad [30]. In turn, Attoui and Bouzid [31] provided a method of modelling gaskets using a structural mechanical model.

The micromechanical approach is a widely used technique for the analysis of composite materials. A spiral wound gasket, which is made of metal rings for stability and filler that predominates in sealing performance, can be treated as a composite material. The uniaxial compressive behaviour of spiral wound gaskets from the micromechanical model is in reasonable agreement with experimental values, as shown in [5]. A better gasket stress distribution can be obtained using the micromechanical model, assuming that only half of the gasket width is effective for sealing [32]. The sealing behaviour of a flange connection can be increased by effectively enlarging the contact area between the flange and the gasket. Nelson and Siva Prasad [33] proposed a flange connection with a double gasket between the flange interfaces instead of a single gasket. The double-gasket flange connection uses two concentric gasket rings whose individual width is half that of a conventional gasket.

The aim of this paper is to assess the influence of the basic design parameters of spiral gaskets on axial stiffness and the level of tightness. The following parameters were taken into account: the angle of inclination of the central part of the spiral cross-section, the length of the vertical part of the spiral cross-section and the degree of densification of the material filling the metal coils. The scope of work was divided into two stages. The first stage included preliminary tests that involved the determination of the stiffness and tightness of a standard cross-section of a spiral gasket (for two extreme levels of the winding density of the filler), as well as the determination of the elastic–plastic properties of the filler.

In the second stage, multivariate numerical calculations were performed, the purpose of which was to determine the axial stiffness of the gasket as well as to assess the contact pressure distribution on the gasket surface. An innovative aspect of the work was the proposal of a mathematical model that allows the average value of the elasticity modulus of the filler (with regard to the degree of its winding density, i.e., compaction) to be determined. Moreover, the purpose of the research was to conduct an experiment that enables a limited number of analysed variants of models to be assumed in the numerical calculations.

## 2. Materials and Methods

As indicated in the introduction, the basic feature affecting stiffness and tightness is the cross-sectional shape of the spiral part of the gasket and the degree of the winding density of the filler in it. In the first part of the paper, experimental tests were carried out in order to determine the stiffness and tightness of a standard sealing solution that has different degrees of winding density of the filler. Subsequently, tests related to the determination of the elastic properties of the filler, which are necessary to carry out numerical calculations, were conducted. In the final part of the work, numerical calculations were carried out to determine the optimal shape of the cross-section of the spiral part of the gasket.

### 2.1. Experimental Research

#### 2.1.1. Research Object

The tests concerned two solutions of spiral gaskets with a standard spiral cross-section, which differed in terms of the winding degree of the filler. Both variants of the tested gaskets were made in accordance with ASME B16.20 [10], and their dimensions corresponded to the 1½” class 300 designation. The degree of the winding density in the first variant of the gasket design was 0.9 turns/mm of the spiral width, while in the second variant, it was 1.4 turns /mm of the spiral width. The spacer rings (inner and outer) and metal windings of the spiral gaskets were made of 316 L steel. The filler of the spiral gaskets was expanded graphite with a thickness of 1 mm and a density of 1 g/cm^3^.

**Figure 1:**
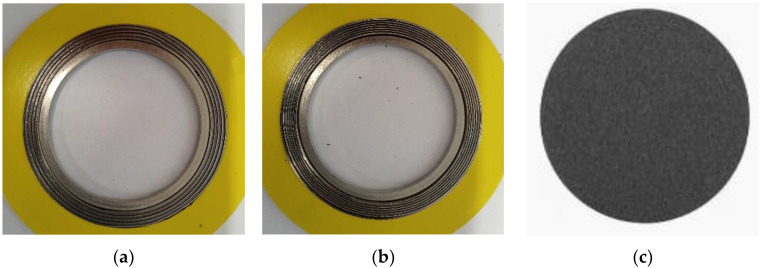
Samples used in the experimental studies: (**a**) first variant of the spiral gasket with graphite filler and a winding density of 0.9 turns/mm; (**b**) second variant of the gasket with a winding density of 1.4 turns/mm; (**c**) disc made of expanded graphite.

For the purpose of further numerical calculations, the elastic–plastic characteristics of the filler were also determined in these tests. The tests involved the compression and the measurement of the axial strain of a disk-shaped sample made of expanded graphite, which had a thickness of 1 mm and a diameter of 70 mm. The samples accepted for the testing are shown in Figure 1.

#### 2.1.2. Test Stand

**Figure 2:**
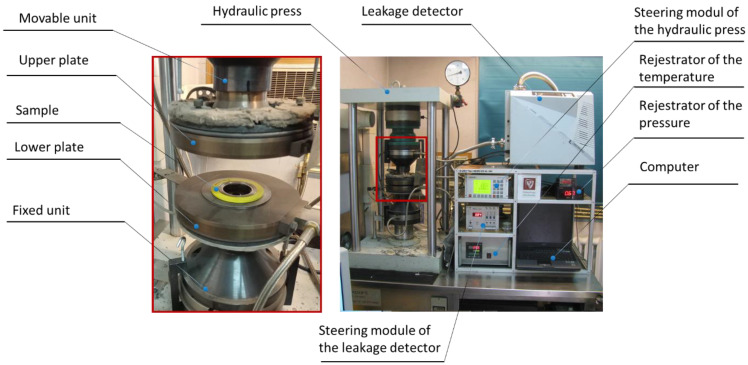
Test stand for the determination of the elastic–plastic characteristics and tightness characteristics of the sealing materials.

The tests related to the determination of the stiffness, tightness, and elastic–plastic properties of the filler were carried out on the test stand shown in Figure 2. This stand consisted of a hydraulic press, the main elements of which included a fixed lower unit and a movable upper unit. The lower unit was equipped with a force sensor, as well as sensors recording the displacement of the upper unit, which in turn measured the degree of compression of the tested sample. The sensitivity threshold of the leakage (helium detector) is 1 × 10^−11^ mbar × L/s, and the maximum error of the detected leakage is no greater than 5%. The sensitivity of the displacement transducer is 1 μm, whereas the force sensor accuracy is 10%.

The displacement of the upper unit was caused by a hydraulic cylinder that was controlled automatically. This solution allowed the press to be fully programmed to implement the given load program for the tested samples. All measurement data, such as compressive force, pressure exerted on the tested sample, or the sample compression, were recorded in the computer memory. The stand auxiliary equipment included a spectrometric helium detector with a vacuum pump that enabled leaks to be measured with regard to the contact pressure exerted on the sample. The pressure inside the tested sample was exerted by the helium that was supplied from the cylinder.

#### 2.1.3. Research Procedures

The procedure related to the determination of the axial stiffness of the gaskets and the determination of the elastic–plastic properties of the filler was carried out in the following steps:

1. Measurements of the geometry of the tested sample (thickness and external and internal diameter). Measurements were made at four points that were evenly distributed around the circumference (every 90 degrees);
2. Central location of a sample on the lower measuring plate of the test stand;
3. Starting the program that controls the displacement of the upper unit of the test stand in order to induce gradual compression of the tested sample; the computer program (controlling the displacement of the upper unit of the test stand) implemented the following load scenario: the compression speed of the sample—5 MPa/min. In the case of the spiral gaskets, the maximum displacement of the top plate of the test stand was 0.9 mm, whereas in the case of testing the filler, the maximum plate pressure (on the surface of the disc) was set as 150 MPa;
4. Simultaneously with the implementation of point 3 of the procedure, the program archived data in the form of the degree of compression of the tested sample and the force or pressure exerted on the surface of the sample;
5. After measuring the compression of the samples, their geometry was remeasured in accordance with point 3 of the procedure.

In the case of the tightness tests, the test procedure was as follows:

1. Measurements of the geometry of the tested sample (thickness and external and internal diameter); measurements were made at four points that were evenly distributed around the circumference (every 90 degrees);
2. Central placement of a sample on the lower measuring plate of the test stand;
3. Placement of the secondary seal that constitutes a collector for measuring the tested medium leaking from the gasket;
4. Starting the program that controls the displacement of the upper plate of the test stand in order to cause gradual compression of the tested sample. The computer program controlling the displacement of the upper platform plate implemented the following load scenario: the compression speed of the sample was 5 MPa/min. The contact pressure at which leakage was measured was 5, 10, 20, 30, 40, 50, 60, 80, 100, 120 and 140 MPa, respectively. At each load point, helium was automatically fed into the gasket at a pressure of 40 bar. The test stand control program automatically measured the leakage using a spectrometric helium detector.

### 2.2. Numerical Calculations

**Figure 3:**
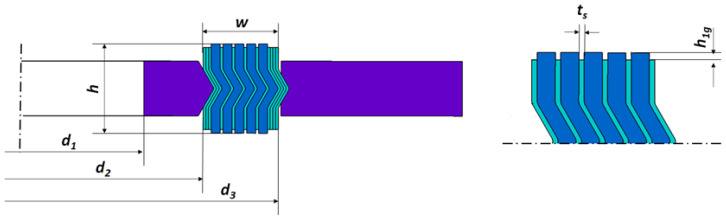
An example of an axisymmetric model of a gasket.

The purpose of the numerical calculations was to determine the basic parameters that describe the shape of the spiral cross-section of the gasket, which affects the optimal sealing solution. The axial stiffness of the gasket was assumed as the optimisation function. According to the guidelines of ASME B16.20 [10], the optimum stiffness of a gasket in the case of 1½” class 300 dimensions should be 120 kN/mm. The constant parameters of the computational models (independent of the gasket profile design variant) included the following: inner diameter of the inner ring *d*_1_ = 48 mm, inner diameter of the spiral *d*_2_ = 54.1 mm, outer diameter of the spiral *d*_3_ = 69.85 mm, total width of the spiral part of the gasket *w* = 7.5 mm, gasket height *h* = 4.45 mm, metal strip thickness *t_s_* = 0.18 mm, and height of the part of the filler strip protruding above the metal strip *h*_1*g*_ = 0.2 mm. A graphic presentation of the parameters describing the geometry of the spiral cross-section is shown in Figure 3.

**Figure 4:**
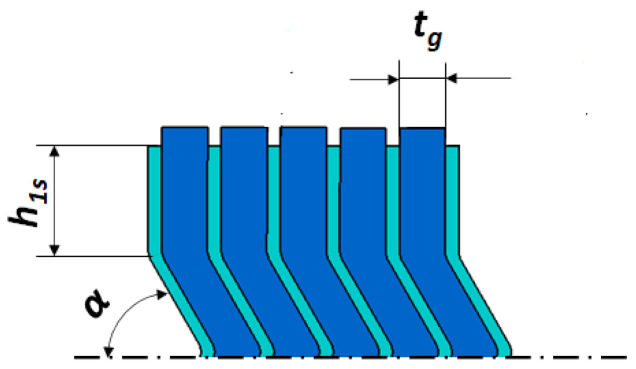
Variables describing the cross-sectional shape of the spiral part of the gasket.

The dimensions of the cross-section of the spiral gasket, which were variable parameters, were as follows: height of the vertical part of the metal strip *h*_1*s*_, angle of inclination of the central part of the spiral *α*, and current thickness of the filler strip *t_g_*. These parameters are shown in Figure 4.

**Figure 5:**
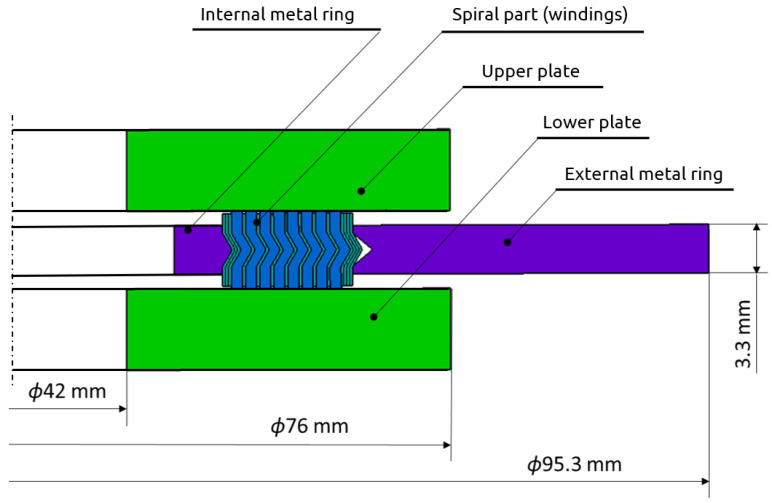
Full geometric model prepared for the numerical calculations.

The fully prepared parametric axisymmetric gasket model that was used for the numerical calculations also included the upper plate and the lower plate of the hydraulic press, which were performing the compression process of the gasket. This model is shown in Figure 5.

In order to determine the minimum number of construction variants that should be adopted for the numerical calculations, an experimental design was used. Due to the three-factor planning space (i.e., three decision parameters), the Box–Behnken experiment design [34,35] was adopted as the most adequate. For the two extreme levels of decision parameters and the three-factor space of the experiment design, the minimum number of calculation variants (according to 2^3^ relationship) is 8. In addition, 3 repetitions will be made in the center of the design space, and therefore, the optimal number of variants of the calculation model was 11. A design of this type is used to determine the second-order response (regression) function in the form:

(1) $$\hat{y}=b_{0}+\sum_{i=1}^{n}b_{i}x_{i}+\sum_{i=1}^{n}b_{ii}x_{i}^{2}+\sum_{i<j}^{n}b_{ij}x_{i}x_{j}$$

whereby:

(2) $$b_{0}=\frac{\sum y_{0}}{3}$$

(3) $$b_{i}=\frac{1}{8}\sum_{n=1}^{11}x_{in}y_{n}$$

(4) $$b_{ii}=\frac{1}{8}\sum_{n=1}^{15}x_{in}^{2}y_{n}-0.0208\left(\sum_{n=1}^{11}x_{1}^{2}y_{n}+\sum_{n=1}^{11}x_{2}^{2}y_{n}+\sum_{n=1}^{11}x_{3}^{2}y_{n}\right)-\frac{\sum y_{0}}{6}$$

(5) $$b_{ij}=\frac{1}{8}\sum_{n=1}^{11}x_{in}x_{jn}y_{n}\left(i\neq j;i=1,\;2,\;3\right)$$

where

$\hat{y}$—response function; in the analysed case, it is the axial stiffness of the gasket;
*x_i_*, *x_j_*—decision parameters (design factors);
*b*_0_, *b_i_*, *b_ii_*, *b_ij_*—polynomial coefficients, which are calculated according to the following dependencies;
$y_{n}$—results obtained from the tests.

Due to the technical possibilities of making a real structure of the spiral gasket, the following ranges of variability of decision parameters were established:

- Height of the vertical part of the profile of metal windings *h*_1*s*_ within the range from 0.8 to 1.6 mm;
- Angle of inclination of the central part of the metal windings α within the range of 45 to 70 degrees;
- Winding density of the spiral gasket *ρ_g_* ranging from 1.18 turns/mm to 1.46 turns/mm; the winding density of the filler determined its thickness after being wound into a spiral.

**Figure 6:**
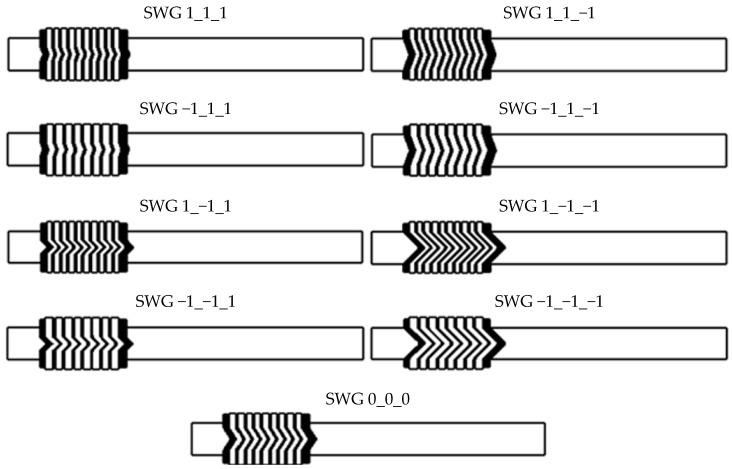
Numbering of the individual variants of the cross-sections of the geometric models.

The determined ranges of the decision variables and their coded (normalised) values are presented in Table 1. The complete matrix of the design is presented in Table 2. The cross-sections of the geometric models, which were generated for the purposes of the numerical calculations (related to the matrix of the experiment design), are shown in Figure 6. The numbering of individual gasket cross-section variants corresponds to the consecutive number from the table showing the matrix of the experiment design. Marking the first variant of the calculation model as SWG 1_1_1 means that the coded variables are set at upper levels—i.e., *x*_1_ = 1, *x*_2_ = 1, *x*_3_ = 1.

**Figure 7:**
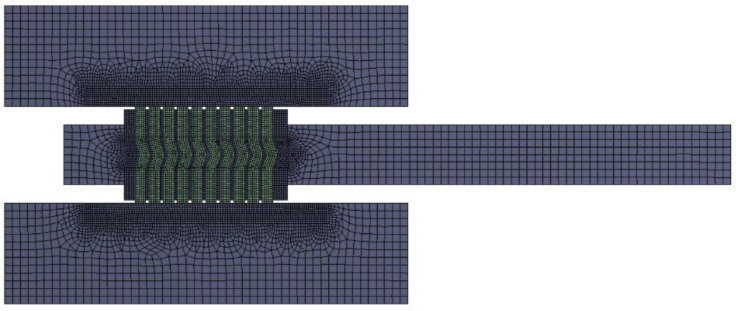
Finite element mesh of the analysed computational model.

Finite elements of the PLANE182 type (with a higher-order shape function) accessible in ANSYS Workbench 19.2 [36] were used to discretise individual geometric models of the analysed construction variants of the gaskets. In particular areas of the model, the mesh of the finite elements was densified, mainly in the area of contact between the gasket and the plates of the hydraulic press. The average dimension of the finite element in the area of the metal windings and filler windings was 0.08 mm. In the case of the spacer rings, the average length of the finite elements was 0.5 mm. At the contact area of the spiral with the plates (upper and lower), the average length of the side edge of the finite element was 0.08 mm. The computational model in this configuration had an average of 28,000 finite elements and 36,000 nodes. An example computational mesh for the SWG 1_1_1 structure variant is shown in Figure 7.

To simulate the mechanical properties of the metal parts of the computational model, material data of 316 L steel with the following elastic–plastic properties were used: longitudinal modulus of elasticity (Young’s modulus) *E* = 180 GPa, Poisson’s ratio *ν* = 0.3, yield strength *R_p_*_02_ = 410 MPa, and plastic hardening modulus of 1.5 GPa. This material was assigned to the following elements of the model: the bottom and top plates of the hydraulic press, the spacer rings (internal and external) and the metal windings of the spiral. In the case of the filler material, a linear elastic model was used, for which the value of the modulus of elasticity (depending on the winding density of the spiral) resulted directly from the tests of the elastic–plastic properties and the developed algorithm presented in Section 3.4. The Poisson’s ratio of this material was assumed to be at the level of *ν* = 0.25.

**Figure 8:**
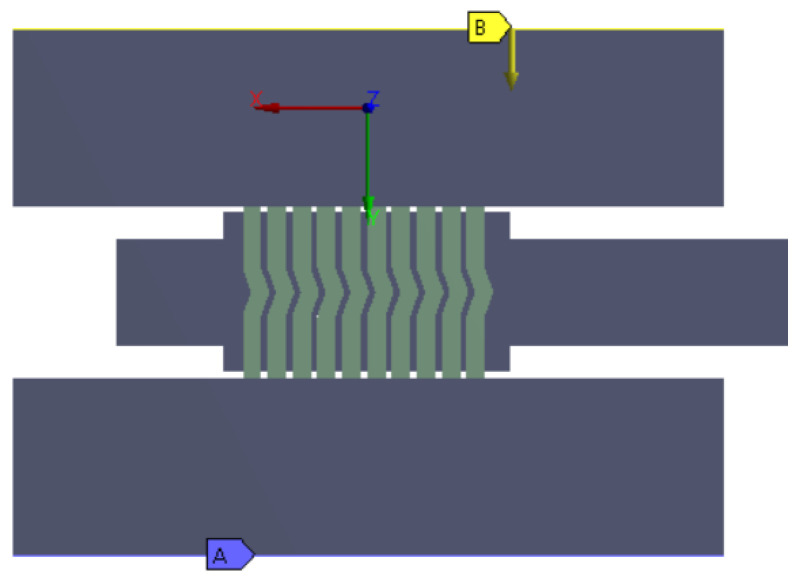
Axisymmetric model of the gasket together with the lower and upper plates of the hydraulic press, which was used in the numerical calculations.

In order to simulate the compression of the gasket, the lower plate was fixed at the base (at the location marked by A), and a gradual displacement was introduced in the upper plate (at the location marked by B), as shown in Figure 8. Friction contact was introduced at the contact area between the surface of the spiral windings and the upper and lower plates. The value of the friction coefficient was set to 0.12 and 0.2, respectively, for the metal windings and the filler windings. The contact area between the metal windings was modelled as being permanently bonded [37].

## 3. Results

### 3.1. Evaluation of the Effect of the Winding Density of the Spiral Gasket on Its Axial Stiffness

**Figure 9:**
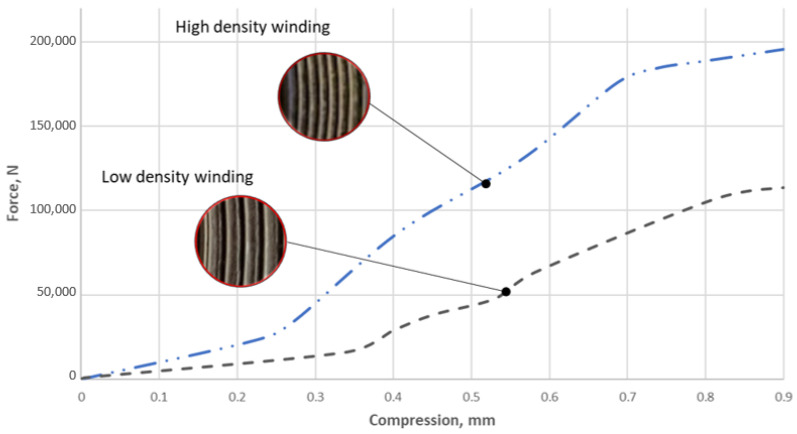
Characteristics of the axial stiffness of the gasket with a low and high degree of winding density.

Figure 9 presents the stiffness characteristics of the tested gaskets, which were obtained on the basis of the tests described in Section 2. These characteristics show the relationship between the axial force exerted on the gasket and the degree of its winding density. At a small compression, i.e., of about 0.05 mm, both characteristics differ slightly. This is due to the fact that in the area of compression (mostly), the graphite strip, which freely protrudes beyond the metal coils, is deformed. Large differences in the course of the curves begin to be noticeable at a compression of above 0.2 mm.

In the case of the gasket with the high-winding density (i.e., 1.4 turns/mm of the gasket width), a 0.3 mm compression required a force of approximately 49 kN, whereas, for the gasket with the low winding density, the same level of deformation was achieved with a force of approximately 17 kN. At the planned maximum compression, i.e., 0.9 mm, the difference in the forces exerted on the gasket was already very significant. In the case of the gasket with the high degree of winding density of the filler strip, the force was equal to 197 kN, whereas in the case of the gasket with the low winding density, it was only 116 kN. It is also worth mentioning that the planned deformation range of 0.9 mm was not accidental, as it resulted from the recommendations of ASME B16.20 [10], which strictly defines the required axial stiffness of a spiral gasket. The standard specifies that with a size of 1½” and a pressure class of 300, a gasket should deform by at least 20% under a contact pressure of 70 MPa. By transferring these values to the dimensions of the gasket, it was calculated that the optimal solution should have an axial stiffness of 120 kN/mm. Taking this into account, and when transferring these values to the graph of the characteristics presented in Figure 9, it can be concluded that the optimal solution of axial stiffness is closer to the gasket with the low degree of winding density of the filler. However, the degree of winding density of the filler is not the only parameter affecting the axial stiffness of a gasket. The cross-sectional shape of the spiral is also of great importance (which was proved, among others, in [38]).

### 3.2. Evaluation of the Influence of the Winding Density of the Spiral Gasket on Its Tightness

**Figure 10:**
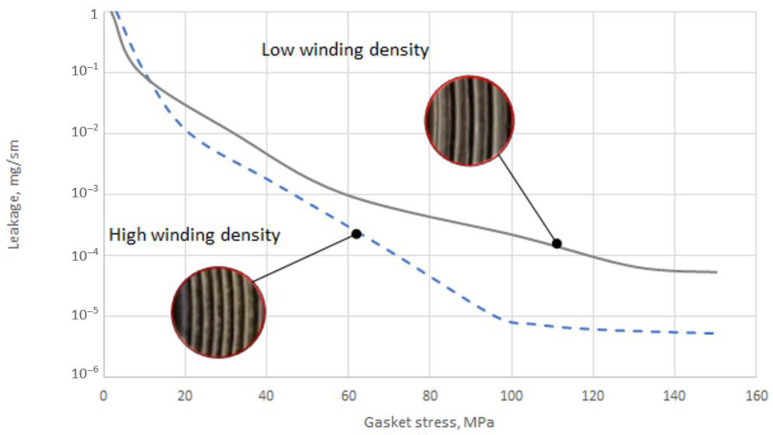
Tightness characteristics of the gasket with regard to the winding density.

Figure 10 shows the tightness characteristics of the two sealing solutions. Within the range from 3 to 10 MPa, both characteristics almost coincide with each other. In the pressure range from 20 to 60 MPa, the sealing characteristics with the high winding density of the filler had a greater tightness.

The increase in tightness by more than an order of magnitude occurs within the pressure range from about 100 MPa to 150 MPa. At the maximum applied contact pressure, the leakage rate of the gasket with the high winding density was equal to 5.3 × 10^−6^ mg/(s × m), and with the low density, it was equal to 5.2 × 10^−5^ mg/(s × m). Moreover, at a pressure of 100 MPa, the gasket with the high winding density of the filler no longer achieves an increase in tightness and stabilises at a constant level. Stabilisation of the leakage in the gasket with the low winding density occurs at a pressure of about 140 MPa.

### 3.3. Elastic–Plastic Properties of the Filler

**Figure 11:**
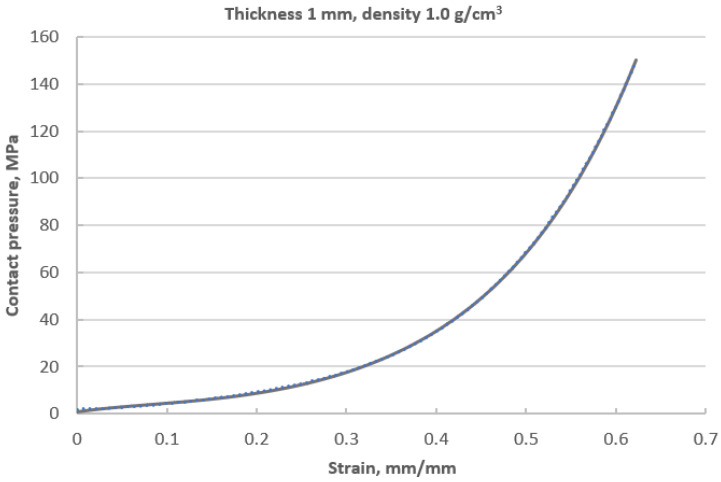
Characteristics describing the contact pressure exerted on the surface of the sample with regard to its axial compression.

Figure 11 shows the compression characteristics of a disc made of expanded graphite. The course of this characteristic is strongly nonlinear and progressive. At the maximum applied pressure of 150 MPa, the sample deformed by 0.62 mm. The value of 150 MPa resulted from the permissible pressure for this material. As can be seen, at a deformation of 0.3 mm, the curve becomes steeper, and the material gradually compacts, which causes a local increase in the average value of the modulus of elasticity. The plotting of these characteristics is necessary in order to determine the local value of the longitudinal modulus. This parameter is the necessary data in order to specify the elastic properties of the material model that is used in numerical calculations. The method of determining the local value of the modulus of longitudinal deformation with regard to the winding density of the filler (presented in this part of the paper) is the leading innovative aspect of research in this area.

### 3.4. Model of the Material of the Filler

**Figure 12:**
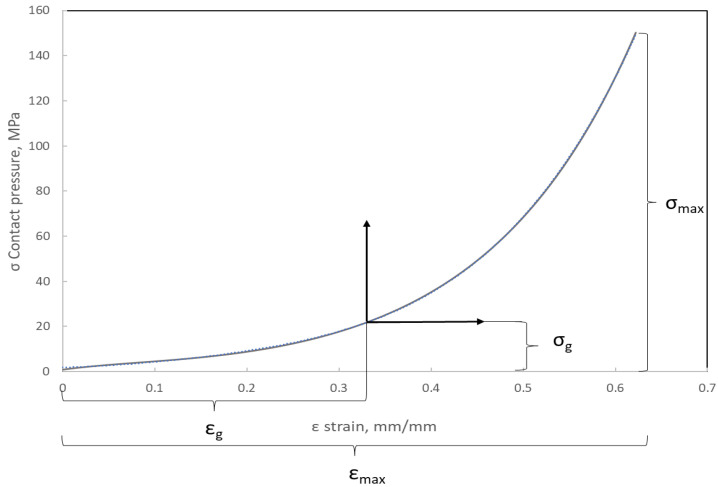
Method of determining the local value of Young’s modulus based on the material compression curve.

Based on the analysis of many papers concerning the numerical modelling of spiral gaskets [22,25,28], it was found that the influence of the degree of winding density of a filler on the axial stiffness has not yet been taken into account. From the analysis of the curve presented in Figure 11, it can be seen that with an increasing compression of the filler, the local value of the modulus of elasticity increases. Indirectly, the value of this parameter determines the angle formed by the line that is tangential to the compression characteristics at the analysed point of the deformation level (Figure 12).

The method of determining the modulus of elasticity of the filler with regard to its deformation is presented below.

**Figure 13:**
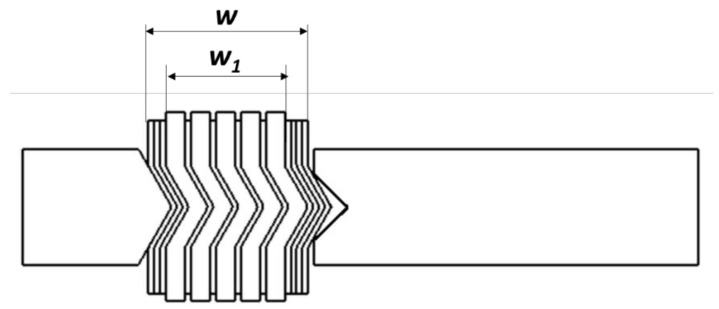
Effective width of the spiral part of the gasket.

Using the geometric relationship of the gasket cross-section (see Figure 13), the effective width of the spiral can be written as follows:

(6) $$w_{1}=w-t_{s}n_{a}$$

where

*w*—total width of the spiral part of the gasket;
*w*_1_—effective width of the spiral part of the gasket (without taking into account the beginning and end windings of the metal strip);
*t_s_*—metal strip thickness;
*n_a_*—total number of the beginning and end windings of the metal strip.

The equation describing the effective width of the gasket can also be presented in another way:

(7) $$w_{1}=t_{g}n_{g}+t_{s}\left(n_{g}-1\right)$$

where

*t_g_*—current thickness of the filler strip;
*n_g_*—total number of windings of the filler.

On the basis of Equation (7), the number of windings of the filler can be determined with regard to the other cross-section parameters, i.e., the thickness of the metal and filler metal strips and the effective width of the gasket. The transformation of Equation (7) results in the following relation:

(8) $$n_{g}=\frac{\left(w_{1}+t_{s}\right)}{\left(t_{g}+t_{s}\right)}$$

In turn, the winding density of the spiral gasket can be expressed as the ratio of the number of windings of the filler to the effective width of the gasket:

(9) $$\rho_{s}=\frac{n_{g}}{w_{1}}$$

The unit of this parameter will be the number of turns/mm of the width of the spiral gasket. After introducing Equations (7) and (8) into Equation (9), the following is obtained:

(10) $$\rho_{s}=\frac{w_{1}+t_{s}}{\left(t_{g}+t_{s}\right)\left(w-t_{s}n_{g}\right)}$$

By transforming (10), an equation describing the current thickness of the filler with regard to the winding density of the spiral can be obtained:

(11) $$t_{g}=\frac{w-t_{s}\left(n_{a}-1\right)-\rho_{s}(w-t_{s}n_{a})}{\rho_{s}(w-t_{s}n_{a})}$$

By relating the current width of the filler to its initial thickness, the degree of deformation can be calculated using the following:

(12) $$\varepsilon_{g}=\frac{∆t_{g}}{t_{g0}}$$

or by using the following:

(13) $$\varepsilon_{g}=1-\frac{t_{g}}{t_{g0}}$$

where

*t_g_*_0_—nominal thickness of the filler strip (thickness before being wound into a spiral);
Δ*t_g_*—compression of the filler.

**Figure 14:**
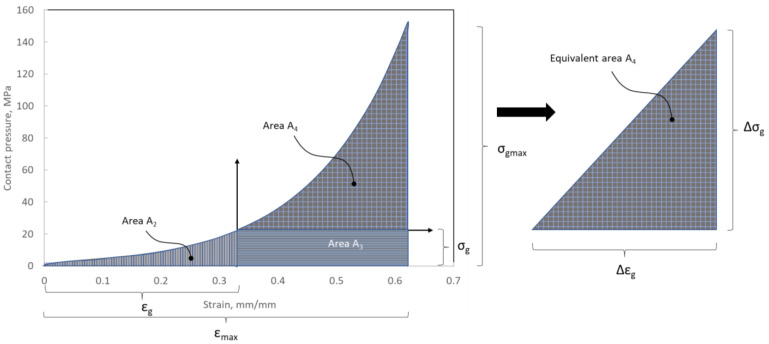
Graphical presentation of the surface area under the curve of the filler compression, which represents the local value of the modulus of elasticity.

The deformation of the filler described by Equation (12) or (13) allows the location of the point on the compression curve (Figure 12) to be determined and the derivative determining the local value of the modulus of elasticity (with a known analytical notation of the equation that models the course of the compression curve of the filler) to be calculated. In paper [24], a slightly different method of calculating this parameter was proposed, and it was shown that the modulus of elasticity is directly proportional to the area under the stress–strain curve. The modulus of elasticity (calculated in this way) is the average value from the entire deformation range. In the method presented below, the determination of the average value of the modulus of elasticity is based on the model presented in [24] but takes into account the effect of the initial compression/deflection of the material that fills the windings of the spiral gasket. If the filler is initially compressed, the area under the stress–strain curve (representing the local modulus of elasticity) will get smaller. Based on Figure 14, the total area under the curve can be written as the following:

(14) $$A_{1}=\int_{0}^{\varepsilon_{max}}\sigma \left(\varepsilon_{max}\right)$$

Alternatively, after dividing them into characteristic subareas, it can be written as a sum of areas:

(15) $$A_{1}=A_{2}+A_{3}+A_{4}$$

Area *A*_2_ represents the area under the stress–strain curve at the given (initial) compression. The surfaces of this area can be described using the following equation:

(16) $$A_{2}=\int_{0}^{\varepsilon_{g}}\sigma (\varepsilon_{g})$$

*A*_3_ represents the surface area from which the average value of the modulus of elasticity at the initial value of strain is calculated. By transforming Equation (15), the following is obtained:

(17) $$A_{4}=A_{1}-\left(A_{2}+A_{3}\right)$$

In turn, area *A*_3_ is the complement of the total surface area under the curve and can be described by the following relation:

(18) $$A_{3}=\left(\varepsilon_{max}-\varepsilon_{g}\right)\cdot \sigma_{g}$$

After introducing Equations (16) and (18) into Equation (17), the following is obtained:

(19) $$A_{4}=\int_{0}^{\varepsilon_{max}}\sigma \left(\varepsilon_{max}\right)-\left[\int_{0}^{\varepsilon_{g}}\sigma \left(\varepsilon_{g}\right)+\left(\varepsilon_{max}-\varepsilon \right)\cdot \sigma (\varepsilon )\right]$$

The equivalent area of *A*_4_ can be described by the equivalent area of a right triangle with a base of Δ*ε* and a height of Δ*σ*. After transforming these relations, the formula for the height of the side of the triangle is obtained:

(20) $$∆\sigma =\frac{{2A}_{4}}{∆\varepsilon }$$

where Δ*ε* = *ε_max_* − *ε_g_*.

According to the definition of the modulus of elasticity, the following can be written:

(21) $$E\left(\varepsilon \right)=\frac{∆\sigma }{∆\varepsilon }=\frac{2A_{4}}{∆\varepsilon^{2}}$$

After introducing Equations (19) and (20) into Equation (21), the following is obtained:

(22) $$E\left(\varepsilon \right)=\frac{2\left\{\int_{0}^{\varepsilon_{max}}\sigma (\varepsilon_{max}-\left[\int_{0}^{\varepsilon }\sigma \left(\varepsilon \right)+\left(\varepsilon_{max}-\varepsilon \right)\cdot \sigma (\varepsilon )\right]\right\}}{∆\varepsilon^{2}}$$

After taking into account Equation (13), Equation (22) can be written in the form:

(23) $$E\left(t_{g}\right)=\frac{2\left\{\int_{0}^{\varepsilon_{max}}\sigma \left(\varepsilon_{max}\right)-\left[\int_{0}^{\varepsilon }\sigma \left(\varepsilon \right)+\left(\varepsilon_{max}-\left(1-\frac{t_{g}}{t_{g0}}\right)\right)\cdot \sigma (\epsilon )\right]\right\}}{{\left[\varepsilon_{max}-\left(1-\frac{t_{g}}{t_{g0}}\right)\right]}^{2}}$$

Equation (23) allows the current (average) value of the modulus of elasticity of the filler of the spiral gasket with regard to its current thickness to be determined. Indirectly, the thickness of the filler can be described with the use of the parameter that determines its winding density. The calculated values of the average value of the modulus of elasticity (using Equation (23)) with regard to the thickness of the filler are presented in Table 3.

### 3.5. Results of the Numerical Calculations

**Figure 15:**
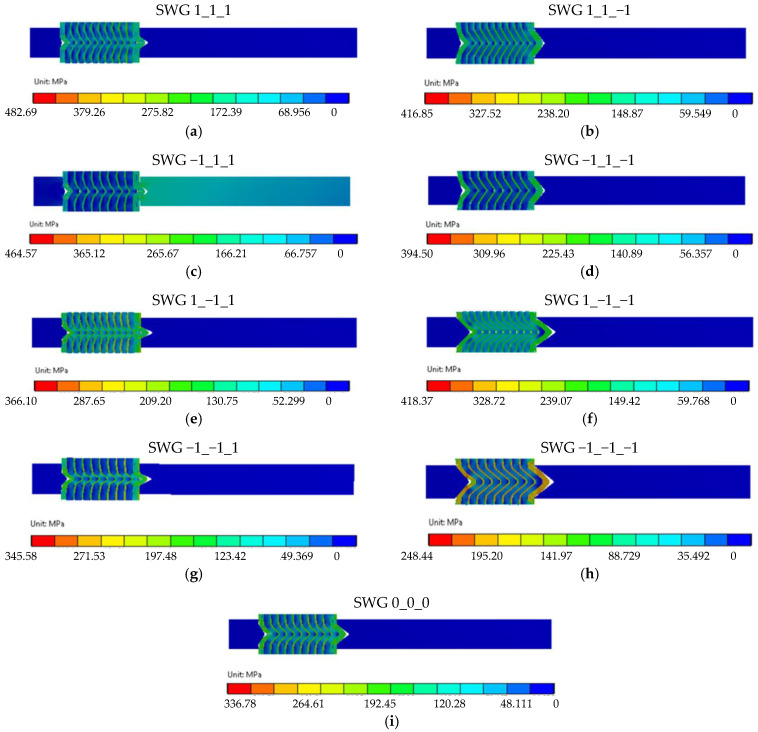
Maps of the reduced stress distribution for nine cases of the analysed gasket structures.

Figure 15 shows the results of the numerical calculations in the form of a map of the stress distribution in the gasket cross-section for the nine analysed variants of the structure. Figure 15a–i show the stress distribution in the case of the maximum compression of 0.9 mm (20% of strain).

**Figure 16:**
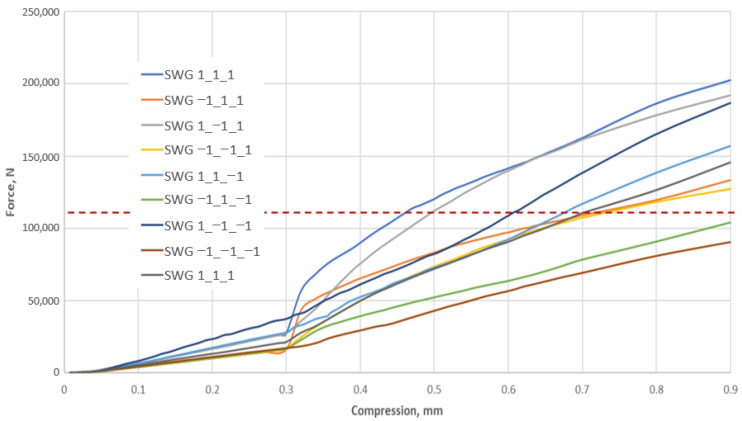
Stiffness characteristics of the nine variants of the gasket construction.

In all the variants of the structure, the maximum stress appears within the area of the metal windings of the spiral gasket and ranges from 482 MPa (for the SWG 1_1_1—see Figure 15a) to 248 MPa (for the SWG −1_−1_−1—see Figure 15h). It can, therefore, be concluded that the parameters that determine the shape of the spiral profile (which are set at the upper levels) cause the highest maximum stress in the metal windings, whereas a low level of these parameters causes the lowest maximum stress value. The distribution of stress in the filler windings is slightly less intuitive. In this case, the highest value of the maximum stress (amounting to 220 MPa) appears in the SWG 1_−1_−1 variant—see Figure 15f. In turn, the smallest value of the maximum stress of these windings appears in the SWG −1_1_−1 variant—see Figure 15d, and is equal to 28 MPa. The analysis of these values shows that the high winding density and the small angle of inclination of the central part of the metal windings have a decisive influence on the increase in stresses in the filler. However, the stress distribution in the gasket cross-section (both in the metal and filler windings) is not the main purpose of the numerical calculations. The main goal was to obtain an answer to the question of to what extent the decision parameters affect the axial stiffness of the gasket. As known from the previous considerations (Section 3.1), in order to obtain an axial stiffness of 120 kN/mm for a gasket strain of 20 percent, the axial force should be 108 kN. Figure 16 shows the characteristics of the axial force as a function of the compression of the analysed variants of the gasket structure. The SWG −1_1_−1 and SWG −1_−1_1 variants are the closest to the required force value (*F_r_* = 108 kN) for a strain of 20%. These results lead to the conclusion that obtaining the required axial stiffness is fulfilled in the case of a construction variant with a low degree of winding density and a low height of the segment of the vertical part of the turns.

**Figure 17:**
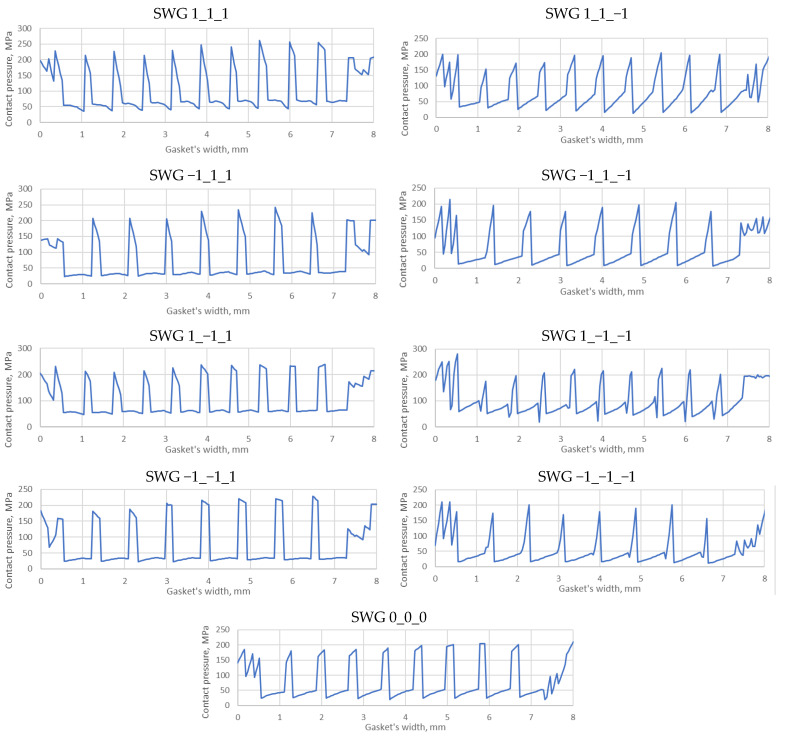
Distribution of the contact pressure for the nine variants of the gasket.

In addition to the required axial stiffness, the analysis of the pressure distribution on the contact surface between the spiral windings and metal plates is also important because the shape of this distribution and its local values directly affect the level of tightness. Figure 17 shows the distribution of the contact pressure in the area of the maximum width of the gasket.

Taking into account the variants that reach the required force (stiffness), i.e., the SWG −1_1_−1 and SWG −1_−1_1 variants, it can be seen that the pressure distribution in the case of the SWG −1_−1_1 variant (both on the metal and filler windings) is more even. In turn, the largest local values appear on the metal windings in the case of the SWG −1_1_1 variant.

### 3.6. Analytical Mapping of the Gasket Stiffness

As emphasised in Section 2, the target result of the numerical calculations was to determine the optimal axial stiffness of the analysed variants of the gasket. A summary of the axial stiffness of the individual construction variants obtained from the numerical calculations, as well as the stiffness obtained using a mathematical model (described by Equation (24)), is presented in Table 4. The values of the individual regression coefficients, which were calculated on the basis of Equations (2) to (5), were as follows: *b*_0_ = 162, *b*_1_ = 39.4, *b*_2_ = 0.1, *b*_3_ = 16.2, *b*_12_ = −5.2, *b*_13_ = −2.1, *b*_23_ = 4.6, and *b*_11_ = *b*_22_ = *b*_33_ = 2.0.

The final form of the mathematical model describing the axial stiffness of the gasket as a function of the decision parameters takes the following form:

(24) $$\begin{array}{c} \hat{y}\left(x_{1},x_{2},\;x_{3}\right)=162+39.4\cdot x_{1}+0.1{\cdot x}_{2}+16.2\cdot x_{3}-5.2{\cdot x}_{1}\cdot x_{2}+\\ -2.1{\cdot x}_{1}\cdot x_{3}+4.6\cdot x_{2}{\cdot x}_{3}+2.0\cdot (x_{1}^{2}+x_{2}^{2}+x_{3}^{2}) \end{array}$$

In order to verify the proposed mathematical model (24), statistical analysis was performed, which included the calculation of the experimental variation, the calculation of the model compliance variation, and the verification of the hypothesis of the acceptance or rejection of the proposed model. The variance of the experiment was calculated using the following relationship:

(25) $$s_{y}^{2}=\frac{\sum_{m=1}^{q}{\left(y_{m}^{n}-\bar{y}\right)}^{2}}{q-1}=262.44$$

where

$\bar{y}$—arithmetic mean of the gasket axial stiffness;
*q*—number of decisive parameters (*q* = 3);
$y^{n}$—axial stiffness of the gasket obtained from the tests.

The only difficulty in this type of analysis is the lack of a statistical error resulting from the numerical calculations. In order to solve this problem and to complete the design matrix, it was assumed that the maximum extreme error of the numerical calculations is equal to 10%. Taking this assumption into consideration, two extreme results were additionally obtained in the middle of the design by decreasing and increasing the value in the middle of the design by 10%.

Variations of the model compliance were calculated as follows:

(26) $$s_{y-\hat{y}}^{2}=\frac{\sum_{l=1}^{N}{\left(y_{l}^{n}-{\hat{y}}_{l}\right)}^{2}}{N-k}=910.5$$

where

$\hat{y}$—response function (axial stiffness of the gasket);
*N*—number of the tests (design variants *N* = 11);
*k*—number of regression coefficients (*k* = 10).

In turn, the hypothesis of accepting or rejecting the model was checked on the basis of the value of the *F*-Sendecor function (*F*-*S*) according to the following dependence:

(27) $$F=\frac{s_{y-\hat{y}}^{2}}{s_{y}^{2}}=3.47$$

If the value of function *F* is lower than the table value for the assumed number of degrees of freedom *v*_1_ = (*N* − *k*) = 1 and *v*_2_ = (*q* − 1) = 2, there is no reason to reject the proposed mathematical model in accordance with [39]. For *v*_1_ = 1 and *v*_2_ = 2, the table value of the statistical function *F*-*S* is equal to 18.513, and therefore, it can be assumed that the model correctly reflects the axial stiffness of the gasket as a function of the decision parameters (with significance level *β* = 0.05).

## 4. Discussion

**Figure 18:**
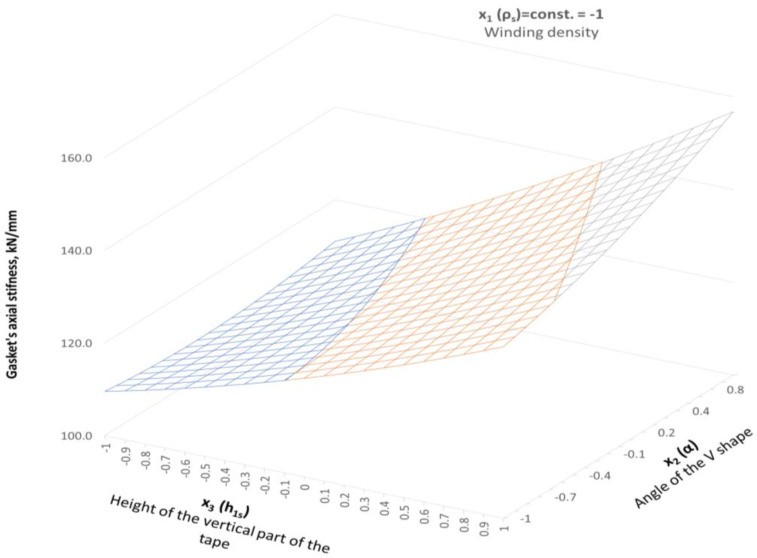
Distribution of the axial stiffness in the gaskets in the case of variable parameters *x*_2_ and *x*_3_, and the constant value of parameter *x*_1_ = −1.

The experimental tests presented in Section 3.1 and Section 3.2 showed that, in the case of increasing winding density, the gasket axial stiffness and tightness increase. The similar dependencies were presented in [13]. The gasket used in that test had a similar construction (the metal strip was made of stainless steel, and expanded graphite with a density 1 g/cm^3^ was used as a filler). In the same way, the gasket density was specified, taking as a density factor the number of turns per one millimetre of the gasket width. The authors specified that a low-density gasket ranged from 0.82 to 1.13 turns/mm, and a high-density ranged from 1.4 to 1.8 turns/mm. It has also been shown that the winding density has a strong impact on the axial stiffness of the gasket, as well as on the leakage rate. In the case of the low-density gasket, at the maximum applied contact stress (86 MPa), the percentage deflection of the gasket was 11%, while the high-density winding gasket deflected about 18%. Similar behaviour was observed in the experimental part of our study, where, at a comparable contact stress of 86 MPa (see Figure 9), the low-density gasket and the high-density gasket deflected by 20% and 11%, respectively. When comparing the effect of gasket winding density on leakage, the authors of the work [13] also proved similar behaviour with the data registered in our study. When the winding density increases, the leakage level decreases. The difference in measured leakage between low-density and high-density gaskets presented in [13] was 10 times greater. In our study, at comparable gasket stress (86 MPa), the high-winding density gasket showed leakage one order of magnitude higher than the low winding density gasket. A novel part of our work was the inclusion of the effect of winding density on the results in the numerical model. The proposed way of determining Young’s modulus strictly depends on the current strip thickness, which directly depends on winding density. With the Box–Behnken experimental plan, the mathematical model describing the axial stiffness of the gasket was developed. Figure 18 shows the distribution of the axial stiffness of the gasket described by the mathematical model according to Equation (24) for variable values of the decision parameters *x*_2_ and *x*_3_ and a constant value of parameter *x*_1_ = −1.

**Figure 19:**
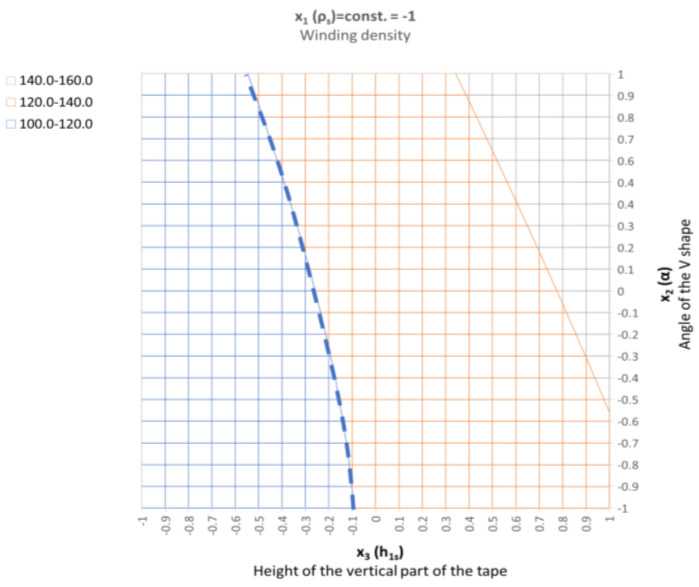
Two-dimensional (2D)—distribution of the axial stiffness in the gaskets in the case of variable parameters *x*_2_ and *x*_3_, and the constant value of parameter *x*_1_ = −1.

Parameter *x*_1_ represents the low winding density, which is equal to 1.18 turns/mm of width. In the case of the parameters being set this way, the stiffness varies from 108 kN/mm to 157 kN/mm, which is within the required optimal range (120 kN/mm). In order to facilitate further analysis, the response surfaces are presented in a two-dimensional form, as shown in Figure 19. It is clearly visible that the stiffness range of the gasket (with the optimal solution) is within the entire range of the variable that describes the angle of inclination of the central part of the spiral profile. In turn, the height of the vertical part, which is described by the *x*_3_ parameter, should be within the range from −0.55 to −0.1.

**Figure 20:**
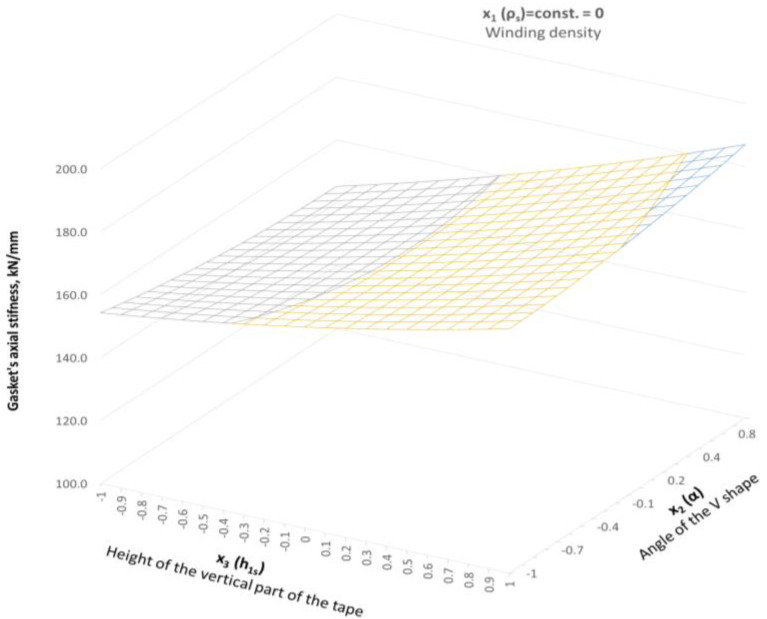
Distribution of the axial stiffness in the gaskets in the case of variable parameters *x*_2_ and *x*_3_, and the constant value of parameter *x*_1_ = 0.

Increasing the winding density of the spiral gasket, even to the level of the average value (within the range of variation of this parameter), results in the stiffness of the spiral part going beyond the expected value of 120 kN/mm. Figure 20 shows the distribution of the axial stiffness of the gasket in the case of a constant value of the winding density equal to 1.32 turns/mm (*x*_1_ = 0).

**Figure 21:**
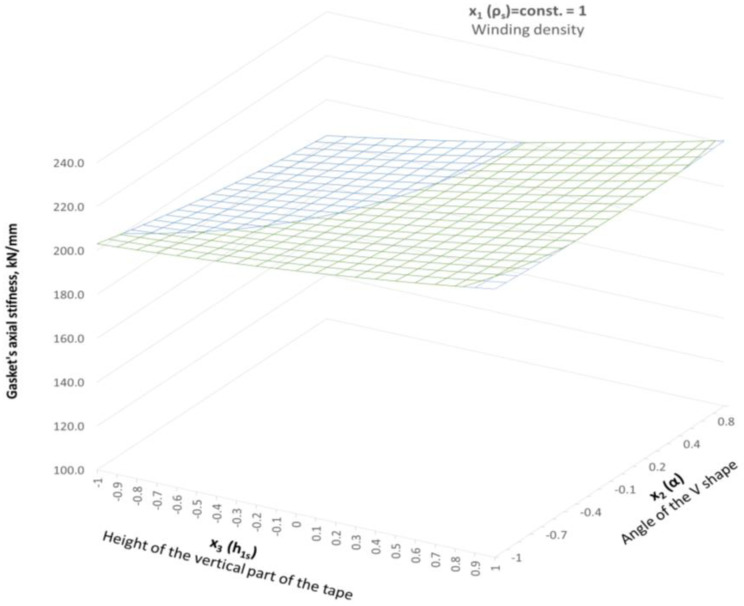
Distribution of the axial stiffness in the gaskets in the case of variable parameters *x*_2_ and *x*_3_, and the constant value of parameter *x*_1_ = 1.

With these settings of the parameters, the gasket stiffness ranges from 145.3 kN/mm to 186.9 kN/mm. In turn, the upper level of the *x*_1_ = 1 parameter means that the axial stiffness of the gasket is in the range from 183.5 kN/mm to 222 kN/mm (see Figure 21).

## 5. Conclusions

The experimental tests showed that the winding density has a significant impact on the compressibility of the gasket (i.e., its axial stiffness) as well as on the leakage level. The winding density can be referred to as the number of turns of the filler strip per one millimetre of the gasket width. In the experimental part of the work, the winding density ranged from 0.9 turns/mm (low winding density) to 1.4 turns/mm (high winding density). For a low winding density gasket, the level of compression at a comparable load is smaller than for a high-winding density gasket. The leakage level at the maximum applied contact stress (150 MPa), in the case of high-winding density, is nearly one order of magnitude higher than in the case of low winding density. A further part of the work showed that as the compression of the expanded graphite filler increases, its modulus of elasticity also increases. Based on the analytical formula proposed by the authors, it is possible to determine Young’s modulus of the graphite strip as a function of the spiral winding density.

Also important is the influence of the shape of the spiral cross-section, described by two decision parameters in the form of the following: the height of the vertical part of the metal strip and the angle of inclination of the central cross-section of the spiral. An increase in the first parameter leads to a decrease in the axial stiffness of the gasket, while the second leads to its increase. The cross-sectional shape of the spiral also affects the contact pressure distribution across the sealing surface. A more uniform distribution of the contact pressure provides better tightness [40].

From the obtained numerical results, it can be stated that the key parameter influencing the axial stiffness of the gasket is the density of the spiral winding, expressed as the number of turns of graphite strip per millimetre of the gasket width. In the lower range of this parameter, at a density of 1.18 turns/mm and for *x*_1_ = −1, achieving the required stiffness can be controlled by selecting the appropriate height of the vertical part of the profile *x*_3_ in the range from −0.55 to −0.1. In turn, the parameter *x*_2_, which is responsible for the angle of inclination of the central part of the spiral profile, can be set within the full range of variation. The adopted method of the experiment plan made it possible to obtain a response function (mathematical model) in the form of the axial stiffness of the gasket depending on three decision parameters governing the shape of the spiral cross-section. The accuracy of this model was verified using three basic criteria: experiment variation, model compliance variation and model acceptance hypothesis. Statistical analysis showed that the mathematical model describes the axial stiffness of the gasket with 99.5% accuracy. This is evidenced by the calculated value of the *F*-Sendecor function.

With the above in mind, the following final conclusions can be drawn:

1. An increase in the winding density leads to a greater axial stiffness of the gasket and, thus, a reduction in its compressibility;
2. As the winding density increases, the gasket tightness increases, and this increase in high contact pressure can be up to an order of magnitude greater compared to a gasket with a lower winding density;
3. The greater the degree of densification of the winding, the greater the stresses created in all elements of the gasket (metal guide rings, steel and filler strips);
4. As the vertical part of the metal strip (parameter *h*_1*s*_) increases, the axial stiffness of the gasket decreases;
5. As the angle of inclination of the central part of the winding cross-section increases, the axial stiffness of the gasket increases.

An innovative achievement of this paper is the mathematical model proposed by the authors that allows to determine Young’s modulus of the filling strip depending on the degree of its densification in the windings, as well as the proposed mathematical model describing the axial stiffness of the gasket depending on the shape of the winding cross-section.

## Figures and Tables

**Table 1:** Coding of independent/decision variables.

Independent Real Variables		
Winding Density of the Spiral Gasket *ρ_s_*, turns/mm	Angle of Inclination *α*, Degrees	Height of the Vertical Part of the Metal Strip *h*_1*s*_, mm
1.18	45	0.8
1.46	70	1.6
1.32	57.5	1.2
0.14	12.5	0.4
Independent coded variables		
*x*_1_	*x*_2_	*x*_3_
−1	−1	−1
1	1	1
0	0	0

**Table 2:** Experiment design matrix for the second-order response function model.

Geometric Model Variant	Test No.	Design Matrix						
		*x*_0_	*x*_1_	*x*_2_	*x*_3_	*x*_1_ *x*_2_	*x*_1_ *x*_3_	*x*_2_ *x*_3_
SWG 1_1_1	1	1	1	1	1	1	1	1
SWG −1_1_1	2	1	−1	1	1	−1	−1	1
SWG 1_−1_1	3	1	1	−1	1	−1	1	−1
SWG −1_−1_1	4	1	−1	−1	1	1	−1	−1
SWG 1_1_−1	5	1	1	1	−1	1	−1	−1
SWG −1_1_−1	6	1	−1	1	−1	−1	1	−1
SWG 1_−1_−1	7	1	1	−1	−1	−1	−1	1
SWG −1_−1_−1	8	1	−1	−1	−1	1	1	1
SWG 0_0_0	9	1	0	0	0	0	0	0

**Table 3:** Values of the longitudinal elasticity modulus of the filler with regard to the degree of winding density of the spiral gasket.

Winding Density of the Spiral Gasket *ρ_s_*, Turns/mm	Current Thickness of the Filler *t_g_*, mm	Young’s Modulus *E*(*t_g_*), MPa
1.18	0.69	290.9
1.32	0.59	420.5
1.46	0.52	544.9

**Table 4:** Summary of the axial stiffness obtained on the basis of the numerical and analytical calculations.

Experiment No.	Geometric Model Variant	FEM, *y^n^*	Response Function, $\hat{y}$
1	SWG 1_1_1	225.3	221.0
2	SWG −1_1_1	148.0	156.8
3	SWG 1_−1_1	213.3	222.1
4	SWG −1_−1_1	141.3	137.0
5	SWG 1_1_−1	174.7	183.5
6	SWG −1_1_−1	115.3	111.0
7	SWG 1_−1_−1	207.3	203.0
8	SWG −1_−1_−1	100.7	109.5
9	SWG 0_0_0	162.0	162.0
10	SWG 0_0_0	178.2	162.0
11	SWG 0_0_0	145.8	162.0

## Data Availability

Data are available upon request from the corresponding author.

## Nomenclature

**Symbol**	**Definition**	**Unit**
*A*_1_, *A*_2_, *A*_3_, *A*_4_	Characteristic areas	mm^2^
*b*_0_, *b_i_*, *b_ii_*, *b_ij_*	Polynomial coefficients	-
*d*_1_, *d*_2_, *d*_3_	Characteristic gasket diameters	mm
*E*	Young’s modulus	MPa
*F*	*F*-Sendecor function	-
*h*	Gasket height	mm
*h*_1*g*_	Height of the part of the filler strip protruding above the metal strip	mm
*h*_1*s*_	Height of the vertical part of the metal strip	mm
*k*	Number of regression coefficients	-
*n_a_*	Total number of the beginning and end windings of the metal strip	-
*n_g_*	Total number of winding of the filler	-
*N*	Number of the tests	-
*q*	Number of decisive parameters	-
*R_p_*_0.2_	Yield strength	MPa
$s_{y}^{2}$	Variance of the gasket axial stiffness experiment	kN/mm
$s_{y-\hat{y}}^{2}$	Variance of the model compliance	kN/mm
*t_s_*	Metal strip thickness	mm
*t_g_*	Current thickness of the filler strip	mm
*t_g_*_0_	Nominal thickness of the filler strip	mm
*w*	Total width of the spiral part of the gasket	mm
*w*_1_	Effective width of the spiral part of the gasket	mm
$\hat{y}$	Response function (axial stiffness of the gasket)	kN/mm
*y^n^*	Axial stiffness of the gasket obtained from the tests	kN/mm
$\bar{y}$	Arithmetic mean of the gasket axial stiffness	kN/mm
*α*	Angle of inclination of the central part of the spiral section	°
*β*	Level of significance	-
Δ*t_g_*	Compression of the filler	mm
*ε*, *ε*_g_, *ε*_max_	Strain, current strain of the filler, maximum strain of the filler	-
*ν*	Poisson’s ratio	-
*ν*_1_, *ν*_2_	Numbers of degrees of freedom	-
*ρ_s_*	Winding density of the spiral gasket	turns/mm
*σ*, *σ*_g_, *σ*_max_	Stress, current stress of the filler, maximum stress of the filler	MPa
*ϕ*	Diameter	mm
